# Cellular Catabolism of the Iron-Regulatory Peptide Hormone Hepcidin

**DOI:** 10.1371/journal.pone.0058934

**Published:** 2013-03-11

**Authors:** Gloria Cuevas Preza, Rogelio Pinon, Tomas Ganz, Elizabeta Nemeth

**Affiliations:** 1 Department of Dermatology, Keck School of Medicine, University of Southern California, Los Angeles, California, United States of America; 2 Department of Medicine, David Geffen School of Medicine, University of California, Los Angeles, California, United States of America; 3 Department of Pathology, David Geffen School of Medicine, University of California, Los Angeles, California, United States of America; National Institute of Child Health and Human Development, United States of America

## Abstract

Hepcidin, a 25-amino acid peptide hormone, is the principal regulator of plasma iron concentrations. Hepcidin binding to its receptor, the iron exporter ferroportin, induces ferroportin internalization and degradation, thus blocking iron efflux from cells into plasma. The aim of this study was to characterize the fate of hepcidin after binding to ferroportin. We show that hepcidin is taken up by ferroportin-expressing cells in a temperature- and pH-dependent manner, and degraded together with its receptor. When Texas red-labeled hepcidin (TR-Hep) was added to ferroportin-GFP (Fpn-GFP) expressing cells, confocal microscopy showed co-localization of TR-Hep with Fpn-GFP. Using flow cytometry, we showed that the peptide was almost completely degraded by 24 h after its addition, but that lysosomal inhibitors completely prevented degradation of both ferroportin and hepcidin. In addition, using radio-labeled hepcidin and HPLC analysis we show that hepcidin is not recycled, and that only degradation products are released from the cells. Together these results show that the hormone hepcidin and its receptor ferroportin are internalized together and trafficked to lysosomes where both are degraded.

## Introduction

The interaction between the iron-regulatory hormone hepcidin and its receptor, the cellular iron exporter ferroportin, is central to mammalian iron homeostasis. Hepcidin is a 25 amino acid hepatic peptide hormone that controls the delivery of iron to blood plasma so as to meet cellular iron needs but avoid iron excess and toxicity. Hepcidin acts by binding to ferroportin on cell surfaces, inducing ferroportin internalization and degradation, and thereby blocking iron efflux into plasma from professional iron-exporting cells: hepatocytes, duodenal enterocytes, splenic and other macrophages and syncytiotrophoblasts [1], [2]. Dysregulated or defective production of hepcidin and ferroportin, or a defect in their interaction, results in various iron disorders.

Many peptide hormones, including insulin, parathyroid hormone and substance P, induce receptor endocytosis. Endocytosed receptors may be degraded or recycled but their ligands may dissociate during endocytosis and thus may or may not follow the fate of their receptor [3]. Previous studies have shown that binding of hepcidin to ferroportin results in ubiquitination of the receptor which leads to its internalization and subsequent degradation in lysosomes [1], [4]–[6]. Although the pathways of ferroportin internalization and degradation have been partially characterized, the molecular mechanisms governing the intracellular fate of hepcidin have not yet been investigated. Here we examine the formation of the hepcidin-ferroportin receptor-ligand complex and follow the cellular fate of the receptor-bound hepcidin.

## Materials and Methods

### Cell line

ECR293-Fpn, a stable cell line expressing Fpn-GFP under the control of ponasterone-inducible promoter was described previously [1].

### Fluorescent hepcidin preparation

Texas-red hepcidin (TR-Hep) preparation was previously described [7]. Briefly, synthetic hepcidin (Peptides International, Louisville, KY) was incubated with Texas Red-X succinimidyl ester (Molecular Probes, Eugene, OR) in PBS with sodium bicarbonate for 1 hr at RT. Labeled hepcidin was purified on a C18 Sep-Pak cartridge (Waters, Milford, MA), followed by the purification by RP-HPLC on a Vydac C18 column (Waters). Purified TR hepcidin was resuspended in DMSO and stored protected from light. Each time a working solution of TR-Hep was prepared, the solution was vigorously resuspended and sonicated to disrupt peptide aggregates.

### Confocal microscopy analysis

ECR-Fpn cells were plated in a 12-well plate on 12 mm poly-D-lysine coated round coverslips (Becton Dickinson, Franklin Lakes, NJ) in the presence of 20 µM ferric ammonium citrate (FAC) with or without 10 µM ponasterone. FAC is added to the medium to prevent cellular iron depletion caused by prolonged Fpn-GFP overexpression. After 24 h, ponasterone was washed off, and cells were treated with 1 µg/ml TR-Hep at 37°C protected from light. After 1 hour, cells were washed with PBS two times and the coverslip was placed on a slide, sealed with clear nail polish and immediately analyzed. Images were scanned at the UCLA CNSI Advanced Light Microscopy Facility and the BRI Cell Imaging Facility on a Leica TCS-SP MP Confocal and Multiphoton Inverted Microscope (Heidelberg, Germany) equipped with an argon laser (488 nm blue excitation: JDS Uniphase), a 561 nm (green) diode laser (DPSS: Melles Griot), and a two photon laser setup consisting of a Spectra-Physics Millenia×532 nm green diode pump laser and a Tsunami Ti-Sapphire picosecond pulsed infrared laser tuned at 768 nm for UV excitation. Images were acquired using LCS Lite software (Leica Confocal Software, Leica Microsystems, Manheim, Germany).

### Flow cytometry analysis

ECR-Fpn cells were plated on poly-D-lysine-coated plates in the presence of 20 µM FAC, with or without 10 µM ponasterone. After 24 h, ponasterone was washed off, and cells were loaded with 1 µg/ml TR-Hep for 4 h. After 4 h, cells were washed with PBS, fresh media was added and cells were treated with or without lysosomal inhibitors chloroquine (100 µM) and bafilomycin (100 nM). Twenty hours after the addition of inhibitors, cells were trypsinized and resuspended at 1×10^6^ cells/ml, and the intensity of red and green fluorescence was analyzed by flow cytometry at UCLA Jonsson Comprehensive Cancer Center and Center for AIDS Research Flow Cytometry Core Facility (supported by NIH awards CA-16 042 and AI-28 697, the UCLA AIDS institute, and the UCLA School of Medicine). Flow cytometry was performed on FACSVantage SE sorting flow cytometer with digital electronics (Becton Dickinson, San Jose, CA) and analyzed with FACSDiva software (Becton Dickinson). Cells not induced with ponasterone were used to establish a gate to exclude background fluorescence. Loss of cellular Fpn-GFP was quantified as % of green fluorescence in induced cells not treated with hepcidin. Degradation of cellular TR-Hep was measured as % of red fluorescence in induced cells treated with TR-Hep for 4 h.

### Hepcidin radioiodination

For cellular studies, synthetic hepcidin with M21Y substitution was iodinated with ^125^I as previously described [1].

### Temperature and pH dependent ^125^I-hepcidin internalization assay

^125^I-hepcidin (10^6^ cpm, specific activity ∼10^7^ cpm/µg) was added to ECR-Fpn cells plated on poly-D-lysine coated plates in the presence of 20 µM FAC, with or without 10 µM ponasterone. Cells were incubated with radiolabeled hepcidin at different temperatures (0, 15, 23 and 37°C) for 1 hour. For pH experiments, growth medium was replaced with media adjusted to pH of 2, 4, 5, 6, 7, 8 and 9 (without additional FAC) and radiolabeled hepcidin was added for 1 h at 37°C. Cells were washed with phosphate-buffered saline (PBS) to remove unbound radioactive hepcidin and centrifuged for 2 minutes at 12,000xg through a silicone oil layer (Nyosil M25; Nye). The radioactivity in cell pellets was determined by gamma counting. The temperature-dependence data are expressed in counts-per-minute (cpm), whereas pH-dependence data were normalized because of the variability in absolute cpm values between experiments and expressed as % of radioactivity at pH 7. The radioactivity of uninduced cells subtracted as background for each point.

### Hepcidin recycling

ECR-Fpn cells were plated on poly-D-lysine coated plates in the presence of 20 µM FAC, with or without 10 µM ponasterone, and incubated at 37°C. After 24 h, cells were washed with PBS and 1 ml of serum free media was added. The cells were treated with 5×10^5^ cpm of ^125^I–hepcidin for 2 h (loading period) at 37°C. After 2 h the cells were washed 3 times with DMEM/10% fetal bovine serum to remove unbound ^125^I-hepcidin and 1 ml serum-free media with 20 µM FAC was added to all wells. Cells were incubated at 37°C for 5 min, 4, 8 and 24 h after loading. To prevent lysosomal acidification and proteolysis, 100 nM bafilomycin was added to assigned wells for 24 h. The supernatant was collected, its radioactivity quantified by a gamma counter, and an aliquot subjected to HPLC analysis. The cells were washed once and centrifuged for 2 min at 12,000xg through a silicone oil layer (Nyosil M25, Nye, Fairhaven, MA). The radioactivity in cell pellets was determined by gamma counting (Beckman, Fullerton, CA).

### HPLC analysis of cell media

Aliquots of the supernatants were analyzed by reversed-phase high-performance liquid chromatography (RP-HPLC) on Vydac C18 column (218TP54; Waters). Fractions were eluted over 30 minutes with an acetonitrile gradient (1 fraction per minute) and their radioactivity determined by gamma counting.

### Statistical analysis

SigmaStat 11 was used for statistical analyses (Systat Software, Point Richmond, CA), and groups were compared using t-test.

## Results

### Effect of temperature and pH on binding/internalization of ^125^I-hepcidin and Fpn

**Figure 1:**
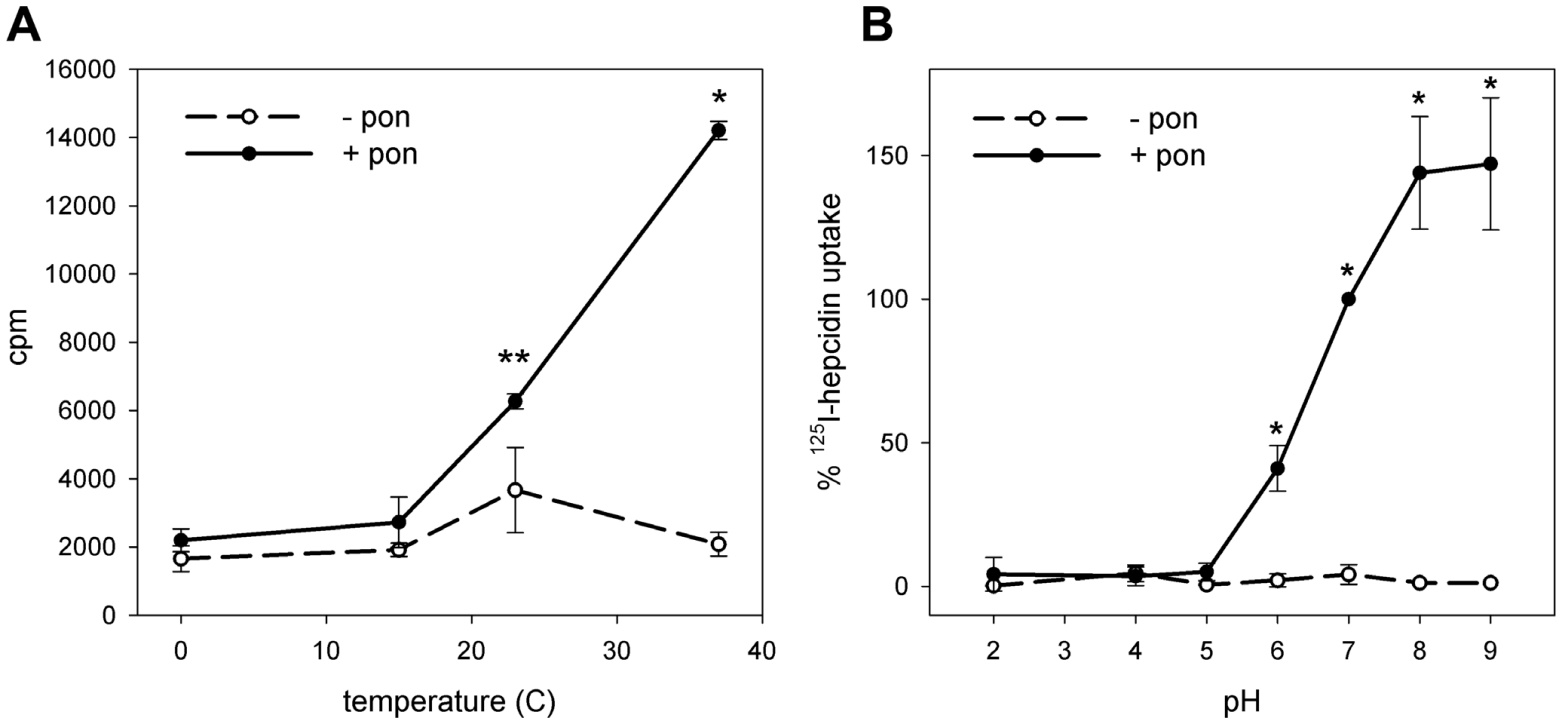
Effect of temperature and pH on the uptake of ^125^I-hepcidin by cells expressing Fpn-GFP. ECR293cells were either not induced (-pon) or induced with ponasterone (+pon) to express Fpn-GFP. (**A**) Cells were incubated with ^125^I-hepcidin for 1 h at 0°C, 15°C, 23°C and 37°C, and the amount of radioactivity bound to cells was measured by gamma counting. *p<0.001, **p = 0.024 compared to uninduced cells at the same temperature. (**B**) Cells were incubated with ^125^I-hepcidin for 1 h at pH 2, 4, 5, 6, 7, 8 and 9, and the amount of radioactivity bound to cells was measured by gamma counter. Radioactivity in uninduced cells was subtracted as background. Because of the variation in absolute counts between separate experiments, data within each experiment were normalized to ^125^I-hepcidin uptake at pH 7. *p<0.01. All experiments were repeated a minimum of three times, and the error bars represent the standard deviation.

The binding of ^125^I-hepcidin to Fpn-GFP expressing cells was temperature- and pH-dependent. Binding decreased with temperatures below 37°C (Figure 1a), confirming our previous observations at 0°C [1]. At 0°C and 15°C, binding was similar in cells expressing or not expressing ferroportin. This temperature dependence may be due to hepcidin insertion into cell membrane during the binding step.

Hepcidin binding to ferroportin was also pH dependent, with 50% binding seen at pH 6 and highest binding at pH 9 (Figure 1b). As disulfide exchange is promoted at alkaline pH, this observation is consistent with our previous work showing thiol-disulfide interaction between hepcidin and ferroportin [7], [8]. Hepcidin did not bind specifically at pH lower than 6.

### Hepcidin is co-internalized with ferroportin

**Figure 2:**
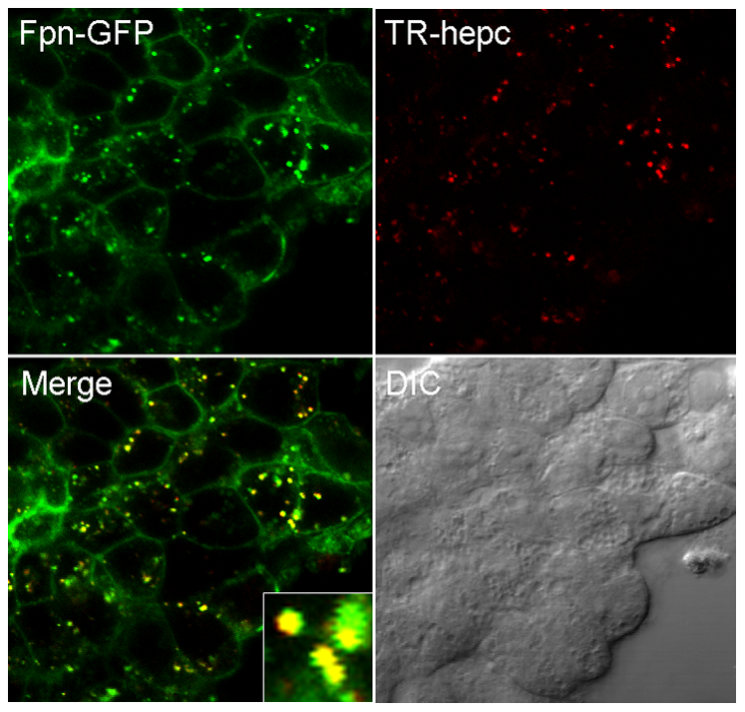
Hepcidin is co-internalized together with ferroportin. TR labeled hepcidin was added to cells expressing Fpn-GFP, and incubated at 37°C for 1 h protected from light. Cells were imaged by fluorescence confocal microscopy. Uninduced cells treated with TR hepcidin had no detectable red fluorescence (data not shown).

To assess the fate of hepcidin after cell surface binding, we added TR-Hep to Fpn-GFP expressing cells for 1 h and using confocal microscopy detected nearly complete co-localization of red hepcidin with green ferroportin in vesicles (Figure 2), indicating that after binding, hepcidin internalizes together with ferroportin.

### Hepcidin is degraded together with ferroportin in lysosomes

**Figure 3:**
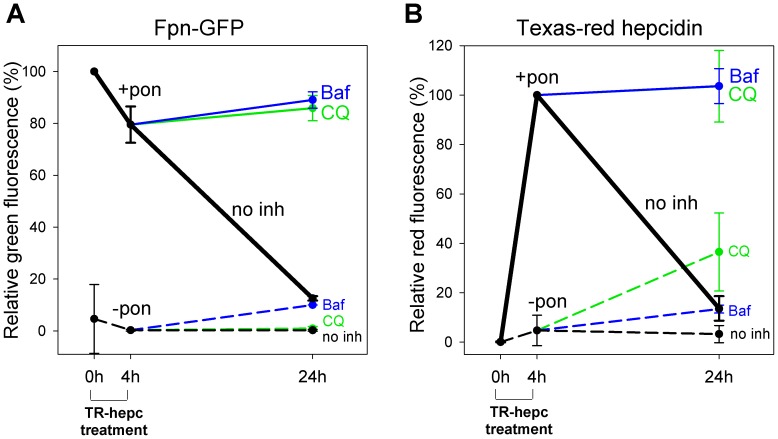
Hepcidin is degraded together with ferroportin. ECR293cells were either not induced (-pon, dashed lines) or were induced with ponasterone to express Fpn-GFP (+pon, solid lines). Cells were treated with Texas Red-labeled hepcidin (TR-hepc) for 4 h, the peptide was then removed, and cells were incubated for another 20 h in the absence or presence of lysosomal inhibitors chloroquine (100 µM, green lines) or bafilomycin (100 nM, blue lines). Cells treated with hepcidin but not inhibitors are represented as black lines (no inh). The amount of cellular Fpn-GFP or Texas Red-hepcidin was determined by flow cytometry. Uninduced cells were used to establish a gate to exclude background fluorescence. (**A**) Quantitation of Fpn-GFP. Data are expressed relative to the green fluorescence of induced cells not treated with hepcidin. (**B**) Quantitation of Texas Red-hepcidin. Data are expressed relative to the red fluorescence of induced cells treated with Texas Red-hepcidin for 4 h.

We previously showed that upon binding of hepcidin, ferroportin is internalized and degraded in lysosomes [1]. Here we used flow cytometry to show that 24 h after addition of TR-Hep to cells, not only Fpn-GFP but also TR-Hep is nearly completely degraded (Figure 3). Addition of lysosomal inhibitors chloroquine and bafilomycin completely prevented the degradation of both hepcidin and ferroportin.

### Hepcidin is not recycled

**Figure 4:**
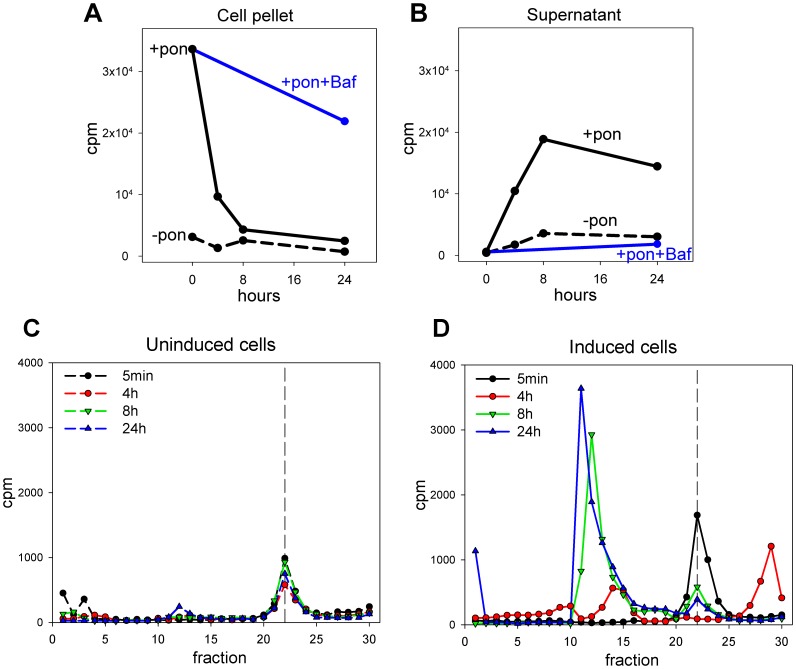
Hepcidin is not recycled. ECR293 cells were either not induced (-pon, dashed lines) or were induced with ponasterone to express Fpn-GFP (+pon, solid lines). ^125^I-hepcidin was added for 2 h to load the cells, excess hepcidin was washed off and fresh media added to the cells. Cell pellets and supernatants were collected after 5 min and 4, 8, and 24 h and radioactivity measured by gamma counting. (A) Counts in cell pellets. (B) Counts in supernatants. (C) and (D) Supernatants harvested at 5 min, 4, 8 and 24 h from uninduced and induced cells respectively were analyzed by reverse-phase HPLC using an acetonitrile gradient (1 fraction per minute). Radioactivity of each fraction was determined by gamma counting. Dashed vertical line indicates the elution fraction of native hepcidin.

In order to determine whether hepcidin is recycled, we added radiolabeled hepcidin for 2 h to cells expressing Fpn-GFP, washed the unbound ligand and monitored the integrity of the radiolabeled species released into the media by HPLC over 24 h. In cells expressing Fpn-GFP, radiolabeled hepcidin was internalized by the cells and the cellular radioactivity was lost over 24 h (Figure 4a). The addition of bafilomycin, an inhibitor of lysosomal acidification and proteolysis, increased the retention of radioactivity in cells, confirming that hepcidin is degraded in lysosomes. Analysis of the supernatants showed that radioactivity was released from cells over 24 h (Figure 4b). To characterize the radioactive species released into the media we analyzed an aliquot of the supernatant by HPLC. In cells not expressing ferroportin, the radioactive species was recovered in a single peak (fractions #21–24) and with low counts (Figure 4c), suggesting that the radioactivity could be attributed to hepcidin that was weakly bound to cell surface and released into the media. In cells expressing Fpn-GFP, the radioactive species recovered from supernatants after 5 minutes also eluted as a single peak in fractions equivalent to those from cells not expressing Fpn (fractions #21–24), suggesting that this species is intact hepcidin. After 4 h, two prominent peaks appeared (fractions # 13–15 and 27–30) and we speculate that these radioiodinated forms could correspond to the two known degradation products of hepcidin-25 detected in urine [9], which are truncated at the N-terminus by 3 or 5 amino acids (hepcidin-22 and hepcidin-20 respectively). The naturally occurring urinary forms eluted from our HPLC system after and before hepcidin-25, respectively [9] and are both much less active than hepcidin-25 [10]. The rest of the molecule is extensively crosslinked by disulfide bonds and would be expected to be more resistant to proteolysis. In supernatants collected after 8 h and 24 h most of the radioactivity eluted in earlier fractions (#10–15), indicative of more extensively degraded forms of hepcidin (Figure 4d).

## Discussion

Our study provides insight into the molecular interactions of hepcidin and ferroportin and establishes the fate of hepcidin after binding to its receptor. Hepcidin binding to ferroportin is temperature-dependent, with no binding seen at 0°C as described before [1], and a progressive increase in binding only at temperatures above 15°C. The temperature-dependent changes in binding are probably not due to conformational changes in hepcidin as the hepcidin conformer populations do not alter sufficiently between 15°C and 37°C to explain differences in biological activity [11]. However, other mechanisms could be involved. Temperature-dependent phase transitions in membrane lipids have been shown to affect transport functions, enzyme activity, and hormone-receptor interactions [12], [13]. Ferroportin was reported to be associated with lipid rafts and alterations of the lipid environment affected hepcidin-induced endocytosis and degradation of ferroportin in macrophages [14]. Moreover, hepcidin is similar to antimicrobial peptides defensins, as shown by its cationic charge, cysteine bridges, and amphipathic structure [11], [15], [16]. Defensins act by embedding in the membrane and increasing membrane permeability [17]. Like defensins, hepcidin has antimicrobial activity, though high concentrations are required for this effect. Given its structural resemblance to defensins, hepcidin may interact with the cell membrane in a temperature-dependent manner, and this interaction could modulate its binding to ferroportin.

Hepcidin binding to Fpn is pH dependent with no binding seen at pHs lower than 6, and the greatest binding at pHs higher than physiological pH (8 and 9). We have previously shown that residue C326 of Fpn is critical for hepcidin binding and that thiols are required for the Fpn-hepcidin interaction, suggesting the possibility of a disulfide bond exchange between one of the four hepcidin disulfide bridges and C326-SH on Fpn [7], [8]. The thiolate anion is the reactive species in a disulfide exchange reaction, so the rate of the reaction is greatest at alkaline pH values [18]. Therefore, higher pH may promote disulfide bond exchange between hepcidin and ferroportin and facilitate stable binding. Other potentially important effects of pH on hepcidin include the effects on histidine in position 3 (H3), an amino acid important for binding to ferroportin [19]. At lower pH, the cationized histidine side chain may decrease ligand-receptor affinity [20].Indeed, substitution of cationic lysine for H3 or several other residues in this region of hepcidin largely ablated hepcidin bioactivity [21].

In this study we show using confocal microscopy that hepcidin and ferroportin co-localize after binding and internalization, and that both are degraded. Addition of the lysosomal inhibitors chloroquine and bafilomycin completely prevented degradation of both molecules, showing that the lysosomes are the main site of endocytic proteolysis for both molecules, and extending on previous reports that the lysosome is the main destination for endocytosed ferroportin [1], [5], [6].

To rule out the possibility that a substantial amount of hepcidin may be recycled, we analyzed radiolabeled hepcidin in the cells expressing Fpn-GFP or not, and their respective supernatants. After a loading period of cells with ^125^I-hepcidin for 2 h, we monitored the integrity of the radioactive species released into the media. There was an almost complete loss of radioactivity from cell pellets after 8 h which correlated with the appearance of radioactive species in the media. HPLC analysis of the supernatant revealed that radioactivity was associated with fractions that eluted at different times than intact ^125^I-hepcidin, indicating that hepcidin is not significantly recycled. In this experiment, addition of bafilomycin, a lysosomal acidification and proteolysis inhibitor, also caused accumulation of radioactivity in cell pellets, confirming that hepcidin is degraded in lysosomes.

This study was the first to examine the fate of hepcidin from its initial binding to ferroportin to its cellular catabolism. We show here that hepcidin is internalized together with ferroportin and that both are degraded in lysosomes. Because of the small size of hepcidin (∼2.7 kD) and its weak binding to plasma proteins [22], glomerular filtration and proteolysis in proximal tubules [23] is thought to be the major mechanism for hepcidin clearance from blood. Detectable concentrations of hepcidin in urine, indicative of peptide that escaped proteolysis in the proximal tubules, are consistent with this mechanism [24], as is the efficient clearance of hepcidin into the dialysate during hemodialysis [25]. The extent to which ferroportin-mediated endocytosis and proteolysis of hepcidin contributes to its clearance under normal or pathological conditions remains to be examined.
